# Triple-negative accessory breast cancer occurring concurrently with primary invasive breast carcinoma: a case report

**DOI:** 10.3389/fsurg.2024.1252131

**Published:** 2024-03-18

**Authors:** Ling Chen, Zujian Wu, Chi Guo, Hengjin Wan, Shouguo Wu, Guoping Wang

**Affiliations:** General Surgery, Tongde Hospital of Zhejiang Province, Hangzhou, Zhejiang Province, China

**Keywords:** accessory breast cancer, binary breast cancer, case report, radical mastectomy, triple-negative breast cancer

## Abstract

**Introduction:**

Accessory breast cancer (ABC) is an extremely rare condition, particularly the presence of triple-negative ABC with ipsilateral invasive *in situ* breast cancer. Binary breast tumors are controversial in terms of surgical methods and comprehensive treatment.

**Case presentation:**

We share the case of a 64-year-old postmenopausal woman who presented with an underarm mass for 3 months. Ultrasonography and computed tomography suggested possible breast cancer with axillary lymph node metastasis. The patient underwent a left modified radical mastectomy combined with axillary lymph node dissection. The postoperative pathology confirmed a binary tumor, prompting us to initiate comprehensive treatment.

**Conclusion:**

We present the treatment approach for a rare case of triple-negative para-breast cancer complicated with carcinoma *in situ* of the breast, hoping to contribute new therapeutic ideas for the treatment of this disease.

## Introduction

The development of mammary tissue typically occurs along the embryonic mammary ridge, which extends from the axilla to the groin area and emerges during the sixth week of gestation (1, 2). While the embryonic mammary ridge usually regresses, except in the breast region where normal mammary tissue forms, there is a possibility for incomplete degeneration of this ridge, leading to the development of accessory mammary glands. During the embryonic period, approximately 90% of accessory mammary glands develop along the mammary ridge, predominantly in the axillary region. However, reports have also documented their occurrence in other areas such as the chest wall, abdominal femoral groove, face, ear, neck, arms, lateral aspect of the leg, and vulva (1, 3). The prevalence of accessory mammary glands in the axilla is estimated to be between 2% and 6% among women (4), with a female-to-male ratio of 5:1 (3).

Accessory mammary development is as hormone-dependent as the normal breast; in other words, any factor that induces breast cancer can also trigger the development of accessory mammary glands. However, accessory breast cancer (ABC) is an extremely rare condition, accounting for approximately 0.2%–0.6% of all breast cancer cases (5). ABC carries a poor prognosis primarily due to early lymph node invasion, high misdiagnosis rates, and the absence of standardized treatment guidelines. The coexistence of ABC and primary invasive breast cancer is exceptionally uncommon. In this report, we present a case study highlighting the diagnosis and management of this unique clinical scenario.

## Case presentation

### Clinical course

The coexistence of ABC and primary invasive breast cancer is exceedingly rare. In this report, we present a case study highlighting the diagnosis and management of this unique clinical scenario. The patient had a history of good health with no underlying medical conditions such as hypertension, diabetes, or heart disease. She was not taking any medications and had no known drug allergies. Menarche occurred at the age of 17 years, and menopause occurred at 54 years old. She experienced regular menstrual cycles and breastfed her son for 1 year. There was no family history of diseases or tumors.

### Physical examination

The patient's left axilla exhibited a palpable mass with a diameter of 2 cm. It displayed a firm texture, indistinct boundaries, adherence to surrounding tissues, and limited mobility. Multiple enlarged lymph nodes were palpable in the left axilla. In addition, several nodules measuring approximately 0.5 cm in diameter were observed in the left breast, while no evident masses were detected in the right breast or right axilla.

### Workup

**Figure 1:**
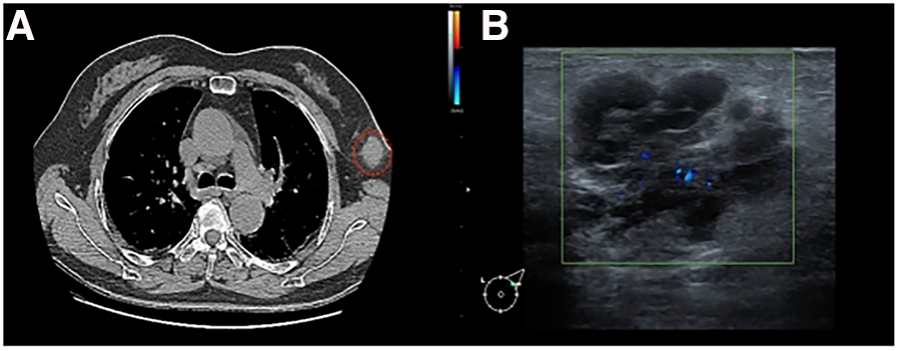
(**A**) Chest CT image: the red circle indicates a mass of the left accessory milk, which is not clearly connected to the breast tissue. (**B**) Mammary ultrasound: A glandular echo with a thickness of approximately 0.8 cm can be seen in the left axilla, and a hypoechoic nodule measuring approximately 3.0 cm × 2.1 cm can be seen on the inside. The boundary is clear and irregular in shape, with early lobulation, and the internal echo is uneven and separated.

The chest computed tomography (CT) image revealed scattered chronic inflammation and fibrous lesions in both lungs, while two low-density soft tissue nodules were observed in the left axilla (Figure 1).

Breast color ultrasound (US) revealed low-echo nodules in both mammary glands. The larger one was approximately 0.4 cm × 0.3 cm (left breast at 3 o’clock) and displayed an oval shape, smooth edges, a horizontal position, and uniform internal echo. A hypoechoic nodule measuring approximately 3.0 cm × 2.1 cm could be observed internally. The boundary was clear and irregular in shape, with early lobulation, and the internal echo was uneven and separated. Color Doppler flow imaging (CDFI) revealed slightly rich blood flow signals within the nodules. Pulsed wave Doppler indicated that the arterial spectrum could be measured, with a resistance index (RI) of 0.7. In addition, lymph nodes measuring 2.1 cm × 0.8 cm were found in the left armpit, showing smooth edges, low echo, and an unclear skin medulla structure. CDFI exhibited slightly rich blood flow signals. In addition, a lymph node echo with a size of 2.9 cm × 0.7 cm was visible, exhibiting a clear boundary and an even internal echo with no visible echo area inside. CDFI exhibited slightly rich blood flow signals. The left axillary accessory mammary gland appeared as hypoechoic nodules, graded BI-RADS 4b, while the left axillary lymph node structure was abnormal (Figure 1).

Based on the medical history and auxiliary examinations, we diagnosed the tumor as an ABC. The TNM classification indicated it as cT2N1M0, corresponding to clinical stage IIb.

### Treatment

**Figure 2:**
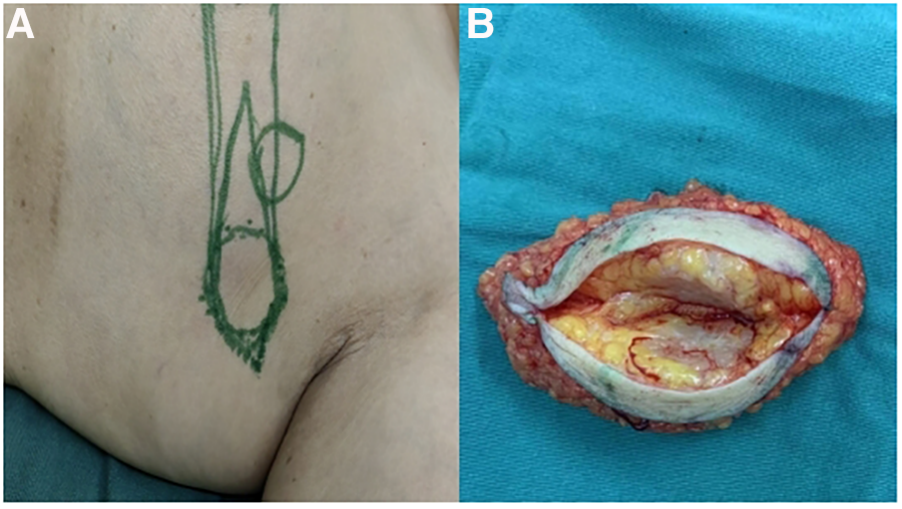
(**A**) Preoperative tumor location markers. (**B**) Postoperative specimen: No definite continuous breast tissue was found in the adipose tissue inside the mass.

After multidisciplinary discussion and considering the patient's tumor clinical stage of cT2N1M0 (clinical stage IIb), combined with the patient's preferences, we performed a modified radical mastectomy for left breast cancer and axillary lymph node dissection. Preoperative tumor location markers and postoperative specimens are shown in Figure 2. In addition to the left ABC, postoperative pathology revealed an invasive ductal carcinoma in the upper inner quadrant of the left breast.

**Figure 3:**
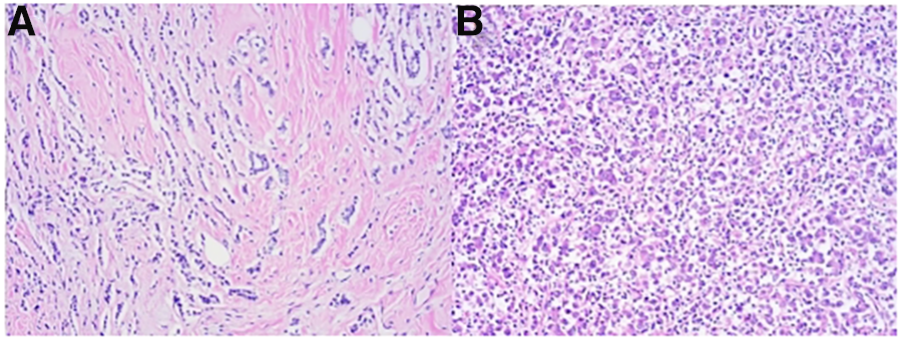
(**A**) Pathology of the left internal upper quadrant mass (invasive ductal carcinoma): non-specific invasive carcinoma *in situ* (approximately 0.5 cm × 1.5 cm, grade II). (**B**) Pathology of the left axillary mass: non-specific invasive breast cancer with medullary features (approximately 2.8 cm × 2.5 cm, grade III).

Postoperative pathology revealed that the left accessory breast mass was a non-specific type of invasive breast cancer with medullary features, measuring approximately 2.8 cm × 2.5 cm, and the histologic grade was characterized as grade III (Figure 3). Immunohistochemical staining revealed the following results: ER and PR expression were negative, p53 was wild-type, and Ki-67 was positive in 90% of cases. Molecular detection results revealed a negative HER2 gene status (no amplification), as identified by fluorescence *in situ* hybridization (FISH), and diploid chromosome 17. Macro-metastasis was found in 5 out of 7 sentinel lymph nodes on the left side, while no cancer metastasis was found in 26 axillary lymph nodes on the left side. The cutting margins, including the basal cutting margin, skin cutting margin, and nipple cutting margin, were all negative. Postoperative pathology showed that the tumor in the medial superior quadrant of the left breast was multifocal invasive ductal carcinoma with a diameter of approximately 0.5–1.5 cm; in addition, carcinoma *in situ* was identified. There was no tumor stromal lymphocyte infiltration, but there was more neurovascular involvement with no intravascular tumor thrombus. The histologic grade was identified as grade II (Figure 3). Immunohistochemical staining results revealed positive expression of ER (3+, 90%), PR (3+, 80%), p53 (wild-type), and Ki-67 (+, 5%). Molecular testing revealed a negative HER2 gene status (no amplification), as identified by FISH, and diploid chromosome 17. The cutting margins, including the basal cutting margin, skin cutting margin, and nipple cutting margin, were all negative. The pathological stage was identified as pT2N1bN0.

According to the results of postoperative pathological analysis and immunohistochemical staining, the patient received postoperative chemotherapy with four cycles of epirubicin and cyclophosphamide, followed by four cycles of docetaxel. The patient has completed all chemotherapy, and there has been no progression of the tumor since surgery.

## Discussion

The accessory mammary gland, also known as an ectopic mammary gland, is the most common breast malformation, which is formed by incomplete degeneration of the mammary gland primordium during the embryonic period (3). The accessory mammary gland shares a tissue structure similar to that of the normal breast; therefore, diseases that can occur in the normal breast can also affect the accessory mammary gland. Any risk factors that may induce breast cancer, such as age, menstruation, fertility, genetics, living environment, and mental factors, cause endocrine system disorders and result in excessive estrogen levels, which can cause ABC. The clinical manifestations of ABC are often the first symptom of an axillary mass or axillary discomfort. Due to the extremely rare nature of this disease in clinical practice, clinicians often treat patients with axillary lymphadenitis or lymphadentuberculosis using routine treatments, which poses difficulty in achieving an early and accurate diagnosis and is prone to clinical misdiagnosis (6).

The treatment principle for ABC is basically equivalent to that of breast cancer, and comprehensive treatment based on surgical treatment is adopted. There is no consensus on the optimal surgical approach for paranasal breast cancer. Madej et al. (7) proposed that unless ABC is very close to or connected with the breast, or cancer cells have been confirmed to invade the side of the breast, there is no need to remove the ipsilateral breast. Evans (8) performed local enlarged axillary tumor resection in addition to axillary lymph node dissection. Compared with radical or modified radical resection, there was no statistically significant difference in survival between the two groups. Drawing on the successful experience of breast-conserving surgery for breast cancer, other experts believe that the scope of surgery for ABC can also be reduced; unless the tumor is very close to the breast or closely connected with the breast, removing the ipsilateral breast is not better than conserving the breast (9). In this case, due to unclear preoperative diagnosis, axillary lymph node metastasis of breast cancer was initially considered, so mastectomy combined with axillary lymph node dissection was performed on the patient. However, the intraoperative frozen section and postoperative pathological analysis revealed the presence of ABC and breast cancer *in situ*. This indicates that clinicians’ understanding of ABC is still insufficient. Therefore, the relationship between the tumor and the surrounding tissue structure can be observed by breast CT, MRI, or even PET-CT before surgery to judge whether the breast has been involved and assess the condition of axillary lymph nodes, which can help in accurately choosing the surgical method to avoid the blindness associated with mastectomy. Although mastectomy is no better than breast-conserving surgery in many cases, the experience gained from this case suggests that if a mastectomy is not performed, particularly careful follow-up is required to rule out any manifestation of a latent primary breast tumor. For some patients with axillary masses appearing fused and fixed, making complete resection during surgery challenging, it is suggested that a coarse needle aspiration biopsy be performed before surgery. If ABC is confirmed, neoadjuvant chemotherapy can be performed first until the tumor shrinks significantly and then surgery can be performed. The clinical characteristics and surgical methods of ABC cases on PubMed over the last decade are shown in Table 1.

**Table 1:** Clinical characteristics and surgical methods of ABC cases on PubMed in the recent 10 years.

Case no.	Reference	Year of publication	Age	Gender	Side	Size (mm)	Surgical method	Follow-up (months)	Outcome
1	(10)	2020	84	M	Right	25	MR	NA	NA
2	(11)	2022	67	M	Right	35	AM	36	NED
3	(12)	2017	64	F	Left	80	MR	NA	NA
4	(13)	2017	87	M	Right	87	AR	24	NED
5	(14)	2022	41	F	Right	5	AM + BCS	12	NED
6	(15)	2022	83	M	Right	30	AM	15	NED
7	(16)	2021	66	M	Left	15	AM	19	NED
			44	F	Left	33	MR + AM	24	NED
8	(17)	2020	43	F	Right	30	AM	NA	NA

F, female; M, male; MR, modified radical mastectomy; AM, accessory mastectomy; BCS, breast-conserving surgery; NA, not available; NED, no evidence of disease.

The principles of postoperative treatment for ABC are basically the same as those for breast cancer. However, because it mostly occurs in the armpit where blood vessels and lymphatic vessels are abundant, early infiltration and metastasis are more likely; thus, the scope of surgical resection will be limited to a certain extent. Therefore, compared with conventional breast cancer, the indications for radiotherapy and chemotherapy can be moderately relaxed in ABC cases (18). Specific chemotherapy regimens can be selected according to the clinical stage and postoperative pathological type of the cancer. Considering that the patient in this case presented with binary cancer, since the breast cancer is *in situ*, our center has established a comprehensive treatment plan mainly for ABC. In this case, ABC is of a triple-negative subtype, so chemotherapy is the main treatment. The patient is currently receiving chemotherapy with four cycles of epirubicin and cyclophosphamide, followed by four cycles of docetaxel. Triple-negative breast cancer (TNBC) patients have been the focus of research due to their poor prognosis, high mortality rates, and invasiveness. Given that triple-negative ABC is extremely rare and has a worse prognosis, our center will proceed with further treatment after standard chemotherapy. The SYSUCC-001 study (18) demonstrated that intensive adjuvant therapy with ciapecitabine following standard treatment outperformed chemotherapy in TNBC patients, bringing significant clinical benefits. Currently, the combination regimen of platinum-containing drugs has also emerged as a major area of research focus in TNBC treatment. Sharma (19) found that a regimen combining docetaxel with carboplatin achieved a higher pathological complete response rate in neoadjuvant chemotherapy for TNBC. These findings provide a new idea for our follow-up treatment, which warrants further exploration. With the further development of precision medicine, a new generation of antibody–drug conjugates, such as T-DXd, has been introduced. Modi et al. (20) suggested that patients with low expression of HER2 could derive significant survival benefits from T-DXd treatment. Immunotherapy has also gained popularity in the treatment of TNBC in recent years due to its significant efficacy. Currently, studies have proved that the anti-PD-1 antibody pabolizumab has a definite effect on the treatment of TNBC (21). The emergence of these studies provides more treatment possibilities for TNBC patients. However, due to the abundance of blood vessels, nerves, and lymphatic vessels in the armpit, different from radiotherapy in the chest wall and supraclavicular region of traditional breast cancer, radiotherapy for ABC has not been studied clearly and is still being explored.

Patients suspected of ABC should be carefully examined to strive for early diagnosis and treatment, and to improve the survival rate of patients. Surgical indications for accessory mammary gland treatment should be strictly mastered, and individualized surgery and comprehensive treatment programs should be formulated for patients to improve their quality of life.

## Conclusion

Due to the rarity and particularity of accessory breast cancer, there is still no clear expert consensus on its standardized treatment. The selection of a surgical program for accessory breast tumors should consider the patient's condition comprehensively and exclude the existence of other tumors fully. We have shared a comprehensive treatment plan for this rare case. We will continue to follow up on the patient's prognosis.

## Data Availability

The original contributions presented in the study are included in the article/Supplementary Material, further inquiries can be directed to the corresponding author.
